# Supplementary figures and images for: A treatment strategy for meeting life as it is. Patients’ and therapists’ experiences of brief therapy in a district psychiatric centre: A qualitative study
Source: PLoS One. 2021 Oct 27;16(10):e0258990. doi: 10.1371/journal.pone.0258990 (PMC8550582; doi:10.1371/journal.pone.0258990)

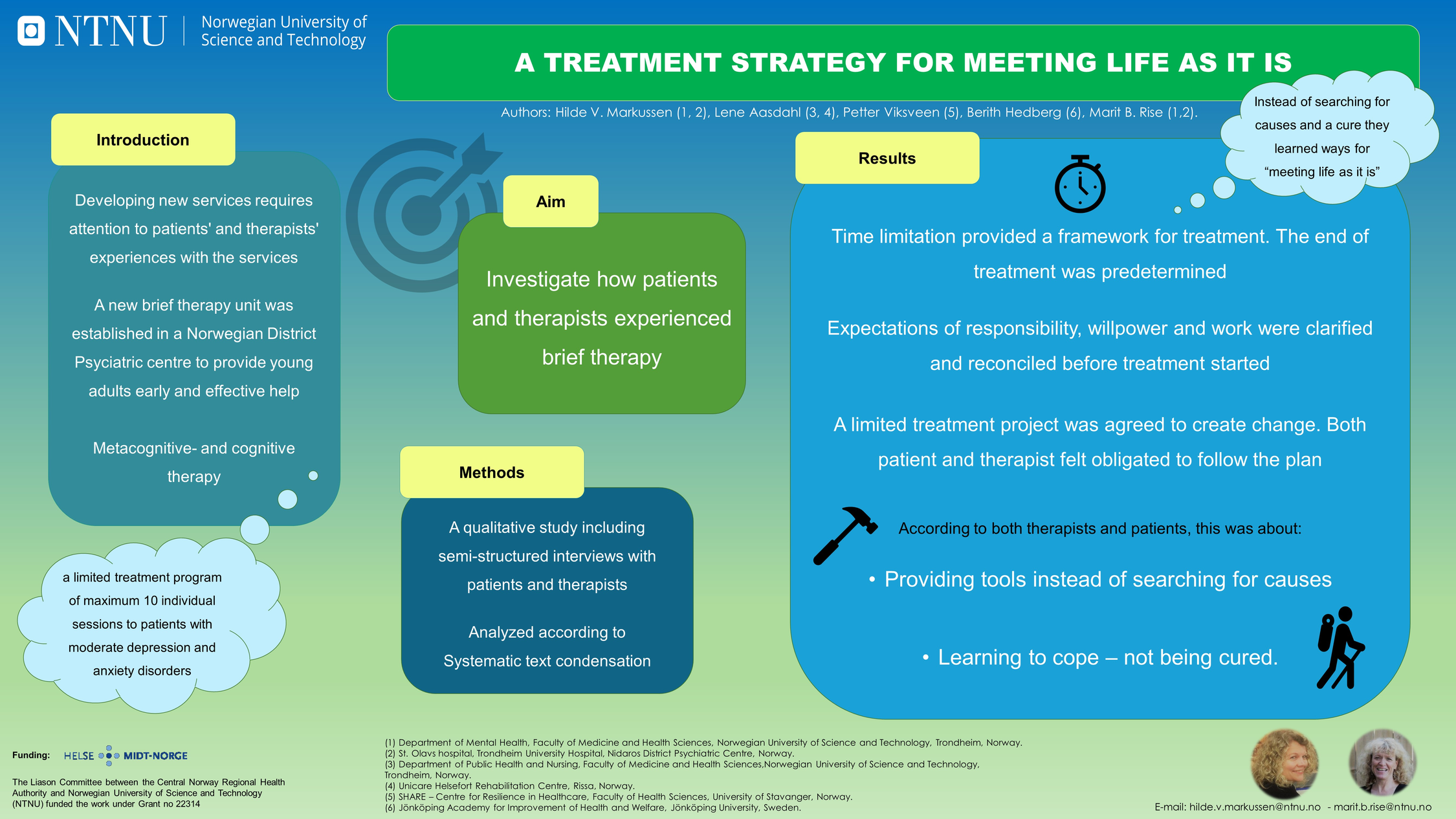

Supplement: S1 Fig — (TIF) [file pone.0258990.s001.tif]
