# Supplementary material for: A treatment strategy for meeting life as it is. Patients’ and therapists’ experiences of brief therapy in a district psychiatric centre: A qualitative study
Source: PLoS One. 2021 Oct 27;16(10):e0258990. doi: 10.1371/journal.pone.0258990 (PMC8550582; doi:10.1371/journal.pone.0258990)
Supplement: Attachment — Submitted filename: 211008 PLOS ONE response letter HVM et al.docx [file pone.0258990.s002.docx]

PLOS ONE

Academic Editor

Frédéric Denis, Ph.D.

Response to reviewers

**PONE-D-21-16580**

**A treatment strategy for meeting life as it is. Patients’ and therapists’ experiences of brief therapy in a district psychiatric centre - a qualitative study**

Dear Editor,

We are grateful for the possibility to resubmit this manuscript after the decision of minor revision. We have incorporated the suggestions from the reviewers and answered all the comments. Please find our responses to the comments below.

Journal requirements:

When submitting your revision, we need you to address these additional requirements.

1. Please ensure that your manuscript meets PLOS ONE's style requirements, including those for file naming.

**Authors’ reply:**

**We have read the guidelines and hope the article is presented as expected. We have used “Calibri” as the theme font, and we have used level 1 and level 2 for headings. You can change the theme font if needed.**

2. Thank you for stating the following in the Acknowledgments/Funding Section of your manuscript:

“The Liaison Committee between the Central Norway Regional Health Authority and Norwegian University of Science and Technology (NTNU) funded the work under Grant no. 22314. The funding body was not involved in the design of the study, the collection, analysis and interpretation of data or in writing the manuscript.”

We note that you have provided funding information that is not currently declared in your Funding Statement. However, funding information should not appear in the Acknowledgments section or other areas of your manuscript. We will only publish funding information present in the Funding Statement section of the online submission form [...] Please include your amended statements within your cover letter; we will change the online submission form on your behalf.

**Authors’ reply:**

**We appreciate the feedback and have updated our Funding statement. We have removed funding information from the manuscript. Our revised text is as follows:**

The Liaison Committee between the Central Norway Regional Health Authority and Norwegian University of Science and Technology (NTNU) funded the work under Grant no. 22314. The funding body was not involved in study design, data collection or analysis, the decision to publish, or the preparation of the manuscript.

The first author (HVM) and the last author (MBR) received the funding through their affiliation with NTNU and St. Olavs Hospital.

**For information:**

**The funder website;** [**https://helse-midt.no/samarbeidsorganet**](https://helse-midt.no/samarbeidsorganet) **and Email: samarbeidsorganet@mh.ntnu.no**

3. In your Data Availability statement, you have not specified where the minimal data set underlying the results described in your manuscript can be found [...] We will update your Data Availability statement to reflect the information you provide in your cover letter.

4. We note that you have indicated that data from this study are available upon request.

In your revised cover letter, please address the following prompts:

a) If there are ethical or legal restrictions on sharing a de-identified data set, please explain them in detail (e.g., data contain potentially sensitive information, data are owned by a third-party organization, etc.) and who has imposed them (e.g., an ethics committee). Please also provide contact information for a data access committee, ethics committee, or other institutional body to which data requests may be sent.

b) If there are no restrictions, please upload the minimal anonymized data set necessary to replicate your study findings as either Supporting Information files or to a stable, public repository and provide us with the relevant URLs, DOIs, or accession numbers. For a list of acceptable repositories, please see http://journals.plos.org/plosone/s/data-availability#loc-recommended-repositories.

We will update your Data Availability statement on your behalf to reflect the information you provide.

**Authors’ reply, 3 and 4:**

**We appreciate that the data availability statement on our behalf will be updated to reflect the information below and are revised as follows;**

**Data availability:**

**There are both ethical and legal restrictions on sharing the data set due to sensitive information.** **It is not possible to publish the original data as participants were guaranteed that their interviews would not be made publicly available. Therefore, data publication would violate their privacy rights and conflict with the General Data Protection Regulation (GDPR).** **The approved information letter to participants in the present study stated; The researchers have a duty of confidentiality, and information from the interviews will not be given to employees at the DPC nor anyone else. This study was approved by the “Regional Committee for Medical and Health Research Ethics” (REK) in central Norway (2018/49). REK approved the information letter before we used it in the study. Email: rek-midt@mh.ntnu.no**

**The committee secretariats can also be contacted by telephone or office address which can be found at the online portal https://rekportalen.no**

(**NB:** We initially indicated that data from this study is available on request, and this statement was intended as an assurance that all data is stored at NTNU and may be checked by REK-approved researchers if needed. Therefore, data from this study should NOT be indicated as “available upon request”.)

**We have removed the following from the Acknowledgments as required in the formatting guidelines;**

**“Competing interests;**

**All authors declare that they have no competing interests.”**

5. Your ethics statement should only appear in the Methods section of your manuscript. If your ethics statement is written in any section besides the Methods, please delete it from any other section. Please review your reference list to ensure that it is complete and correct [...].

1. **Authors’ reply: Our ethics statement now only appear in the Methods section of the manuscript. We have checked the manuscript and can not find errors in the source reference.**

Additional Editor Comments (if provided):

This manuscript aims to explore patients' and therapists' experiences of brief therapy. It is a timely and well-written article that nevertheless raises some minor comments.

**Authors’ reply: Thank you!**

1/P 170 : « The patients reported suffering from depression and/or anxiety, and/or post-traumatic stress disorder ». A medical evalation has it been carried out beforehand ?

**Authors’ reply:** **To elaborate on this, we have added text in the manuscript as follows (Page 8, l.170-172):**

They had been diagnosed with depression and/or anxiety by their GP prior to referral for outpatient treatment at DPC. Additionally, three patients reported also suffering from post-traumatic stress disorder.

**(this is also shown in point 3/ below)**

2/ Could you present the interview grid

**Authors’ reply:** **See reply to reviewer’s response nr five (5) below about adding a Table that summarizes the main findings of the five themes in a structured manner highlighting the main ideas.**

**We have also added the two interview guides in one document as an additional file;**

**Table 1. THE INTERVIEW GUIDES.** **NB: Figure Citation (Fig 1) appears at page 9, l. 192 in the manuscript.**

3/ Please give more information about your participants (age, gender...).

**Authors’ reply: Some information about the participants' age and gender is provided in the manuscript section “Participants and recruitment” from before. We are unable to provide much information on individual participant level as this would provide data that could indirectly lead to an identification of participants and therefore be in conflict with GDPR regulations.**

**However, to elaborate on this, we have added text in the manuscript as follows (Page 8, l. 159-184): Changes are highlighted in bold below (NB; better presented in the “ track changes”-document)**

A total of 20 informants, 12 patients and 8 therapists, took part in the study, participating in individual or focus group interviews. Eligible participants were patients who were currently receiving brief therapy, and therapists who worked at the brief therapy unit. Patients were informed by the therapists about the study while receiving treatment. The therapists provided the first author’s contact information for those who consented to take part. The first author then contacted these patients and made appointments for interviews. Patients were thus recruited through purposeful criterion sampling [28], i.e. those who met the study criteria. Sixteen patients consented to participate. One participant chose to leave the study before the interview took place. Patient interviews were terminated when the authors considered that the study had sufficient information power [29], after **twelve** interviews. The sample **of twelve patients included** seven men and five women, **median age 31 years** (**range** 21 to 47 years), all receiving individual brief therapy at the unit. **They had been diagnosed with depression and/or anxiety by their GP prior to referral for outpatient treatment at DPC. Additionally, three** patients reported **also** suffering from post-traumatic stress disorder. **Half had been diagnosed with more than one condition, and nine out** of **12 had previously received specialized psychiatric treatment. Some reported their** first episode of mental ailments, **whereas** others **had suffered from mental health problems** over several decades. **Thus, although all were diagnosed with depression or anxiety, the sample included patients with different backgrounds and experiences.**

Therapists were recruited through purposeful **criterion** sampling [28], and the inclusion criterion was that they had worked in the brief therapy unit for at least six months. Eight therapists, **two women and six men** were eligible, and all consented to participate in focus group interviews. Their age ranged from 27 to 39 years (**median 35 years**), and their professional background included **clinical psychologists (n=4), psychologists (n=3), and psychiatric nurse (n=1).** **Half of the therapists had several years of experience working** in **the specialist health service, while the others had some experience.** A total of three focus group interviews were conducted, one face-to-face and two by using Skype technology due to the worldwide pandemic outbreak in the spring of 2020.

Reviewers' comments:

Reviewer's Responses to Questions

Comments to the Author

1. Is the manuscript technically sound, and do the data support the conclusions?

Reviewer #1: Yes

2. Has the statistical analysis been performed appropriately and rigorously?

Reviewer #1: Yes

3. Have the authors made all data underlying the findings in their manuscript fully available?

Reviewer #1: No

**Authors’ reply: See Authors’ reply to Journal requirements nr three (3) and four (4) above.**

4. Is the manuscript presented in an intelligible fashion and written in standard English?

Reviewer #1: Yes

5. Review Comments to the Author

Reviewer #1: This is very well written manuscript that explored patients' and therapists' experience of brief therapy, especially how the time frame influences the treatment process, using a qualitative methodology.

I think that the paper brings interesting evidence in addition to the literature.

It would be clearly improved by adding a Table that summarizes the main findings of the five themes in a structured manner highlighting the main ideas.

**Authors’ reply:** **Thank you very much, and we appreciate this suggestion.**

**Table 2, named THEMES WITH KEY ASPECTS, is added as an additional file. Hopefully, the table makes the findings better presented and the main findings more highlighted.**

**NB: Figure Citation (Fig 2) appears at page 12, l. 281 in the manuscript.**

**We have submitted an essential supporting file after the references:**

**As Supporting information is added, a POSTER approved by all co-authors in 2020, which visualize the results from the study. For information, the first author (HVM) has created (she is the owner of the poster and is allowed to present it in PLOS ONE) and presented the poster as preliminary findings to colleagues at** **«ECMH – European Conference on Mental Health 2020» October 1, 2020 (online conference due to covid 19).**

**(The conference website: https://ecmh.eu/)**

6. PLOS authors have the option to publish the peer review history of their article (what does this mean?). If published, this will include your full peer review and any attached files.

Reviewer #1: Yes

While revising your submission, please upload your figure files to the Preflight Analysis and Conversion Engine (PACE) digital diagnostic tool, https://pacev2.apexcovantage.com/. PACE helps ensure that figures meet PLOS requirements. To use PACE, you must first register as a user. Registration is free. Then, login and navigate to the UPLOAD tab, where you will find detailed instructions on how to use the tool. If you encounter any issues or have any questions when using PACE, please email PLOS at figures@plos.org. Please note that Supporting Information files do not need this step.

**The authors' answer: We hope the instructions have been followed. Please contact me if something is missing. (HVM)**

We thank you again for the opportunity to resubmit our manuscript. We look forward to hearing from you.

Trondheim, Norway, October 4, 2021

Yours sincerely

Hilde Våbenø Markussen /s/
